# The outbreak of shigellosis in Sweden as a looming concern for public health: a correspondence

**DOI:** 10.1097/MS9.0000000000000251

**Published:** 2023-02-17

**Authors:** Navneet Kaur, Prakasini Satapathy, Ahmad Neyazi, Bijaya K. Padhi

**Affiliations:** a Department of Geography, Jamia Millia Islamia, New Delhi; Departments of b Virology; c Community Medicine and School of Public Health, Postgraduate Institute of Medical Education and Research, Chandigarh, Punjab, India; d Afghanistan Center for Epidemiological Studies, Herat, Afghanistan

*Dear Editor,*

Since the middle of November, an increasing number of cases of *Shigella* infection with a travel connection to Cape Verde have been reported in Sweden. On 22 December 2022, the Swedish Public Health Agency, Folkhalsomyndigheten, reported 30 cases of shigellosis with a travel connection to Cape Verde that had been reported in Sweden since mid-November. So far, 11 bacterial isolates have been identified: nine *Shigella sonnei* and two *Shigella boydii* 1. European countries such as the Netherlands, Denmark, France, Germany, Portugal, and the United Kingdom have reported cases of *Shigella* infection associated with Cape Verde travel histories^2^. Several countries have taken notice of the spread of infection in Cape Verde and informed the European Centre for Disease Prevention and Control and WHO. Infections with various intestinal pathogens, such as Enterohemorrhagic Escherichia coli, *Campylobacter*, *Cryptosporidium*, and *Giardia*, have also been reported among Swedish travelers. *Shigella* infection associated with travel to Cape Verde has been a persistent issue. This, along with the presence of various species of *Shigella* and intestinal infections, suggests contamination by food.

*Shigella* bacteria are extremely virulent enteric pathogens that inflict shigellosis, an intestinal infection. The most prominent mode of transmission of *Shigella* spp. is fecal-to-mouth contact, which is transmitted from person to person. The main transmission occurs through contaminated food and water. An infected person may experience stomach pain, fever, blood and mucus, and dysentery^3^.

*Shigella* is classified into 43 serotypes and four species or subgroups (A, B, C, and D). Historically, subgroup A is designated as *Shigella dysenteriae*, subgroup B as *Shigella flexneri*, subgroup C as *S. boydii*, and subgroup D as *S. sonnei* 4. Although infections occur worldwide and among people of all ages, prevalent infections are among 1–4-year-olds in low- and middle-income settings. and infected by *S. flexneri* and *S. sonnei* account for most of the infection burden. In the 1970s, a distinct epidemiological niche for *Shigella* became a sexually transmitted disease among men who have sexual relations with other men (MSM). In 2009, uncommon serotypes (*S*. *flexneri* 3a) emerged in England and Wales in the MSM community and expanded across continents among the MSM community to low-risk regions of shigellosis^4^. *S. sonnei* causes travel-associated shigellosis in high-income countries among people who have visited places with a high prevalence of endemic diseases and a periodic upsurge caused by *S. flexneri*. The WHO reports that there are at least 80 million cases of shigellosis per year, with about 700 000 deaths^5^.

Shigellosis contracted during travel can result in the transmission of antimicrobial-resistant *Shigella* to new population groups. The potential for long-term disability from postinfection complexities such as irritable bowel syndrome and reactive arthritis, in addition to the potential of outbreaks in tourist groups and military deployments. During the 18th and 19th centuries, *Shigella* infection was a prevalent and severe disease in Sweden. According to the Annual Report (1998), there were 7078 cases of Shigellosis found in Sweden between 1989 and 1998^6^. Approximately 200–400 cases are reported annually in Sweden, most of which were infected overseas, such as in Turkey, India, and Egypt. *S. sonnei* is the most prevalent species in Sweden now. The number of cases of *Shigella* infection reported in 2008, 2009, and 2015 was 157, 50, and 43, respectively.

In poor hygiene settings, it is very uncommon for infections to spread directly from person to person, and secondary cases are quite common during outbreaks in Sweden. Typically, the shedding period is less than 4 weeks, but substantially longer carriers are possible. Due to the low infectious dose, around 10–100 bacteria are sufficient to cause disease. When bacteria are in the feces through culture, a diagnosis can be determined. The approach is unreliable since bacteria are quickly killed during sample transfer^7^. The cases of *S. sonnei* associated with hotel stays in Cape Verde have so far been confined to European travelers. Consequently, concerns have been raised about the hygiene and sanitation standards of hotels in Cape Verde^8^.

There is no vaccination present. Due to the low infectious dose, the infection can quickly spread from person to person, making strict hygiene imperative. The food industry should be especially aware of the potential for *Shigella* infection. Antibiotics are frequently prescribed both to reduce the symptoms of the disease and to prevent its spread. Along with other hygiene practices, regularly washing of hands with soap and running water can help prevent infection^9^.

Livsmedelsverket, the Swedish food agency, advises travelers traveling abroad, as many countries have higher incidences of disease-causing bacteria and parasites in contaminated food^10^. *Shigella* is a significant cause of worldwide morbidity and mortality, impacting young children in places with few resources disproportionately. An insignificant infectious inoculum and lapses in hygiene and sanitation enable *Shigella* to evade control measures, and the global expansion of strains resistant to current first-line treatments impedes treatment success. The global spread of *Shigella* strains that are resistant to multiple drugs has been fueled by international travel, and since some Shiga toxin-producing strains could be very dangerous, the world desperately needs to be on guard shield^4^.

## Ethical approval

NA.

## Consent

NA.

## Sources of funding

None.

## Author contribution

All authors are equally contributed.

## Conflicts of interest disclosure

None.

## Research registration unique identifying number (UIN)

None

## Guarantor

Ahmad Neyazi.

## Provenance and peer review

Not commissioned, externally peer reviewed.
